# The association of elevated blood pressure during ischaemic exercise with sport performance in Master athletes with and without morbidity

**DOI:** 10.1007/s00421-021-04828-9

**Published:** 2021-10-15

**Authors:** Fabio Zambolin, Jamie S. McPhee, Pablo Duro-Ocana, Bergita Ganse, Liam Bagley, Azmy Faisal

**Affiliations:** 1 Department of Sport and Exercise Sciences, Musculoskeletal Science and Sports Medicine Research Centre, Faculty of Science and Engineering, Manchester Metropolitan University, Manchester, UK; 2 Manchester Metropolitan University Institute of Sport, Manchester, UK; 3 Department of Life Sciences, Musculoskeletal Science and Sports Medicine Research Centre, Faculty of Science and Engineering, Manchester Metropolitan University, Manchester, UK; 4 Saarland University Hospital, Innovative Implant Development, Homburg, Germany; 5 Faculty of Physical Education for Men, Alexandria University, Alexandria, Egypt

**Keywords:** Blood pressure, Master athletes, Sport performance, Ageing, Longevity

## Abstract

**Background:**

An exaggerated exercise blood pressure (BP) is associated with a reduced exercise capacity. However, its connection to physical performance during competition is unknown.

**Aim:**

To examine BP responses to ischaemic handgrip exercise in Master athletes (MA) with and without underlying morbidities and to assess their association with athletic performance during the World Master Track Cycling Championships 2019.

**Methods:**

Forty-eight Master cyclists [age 59 ± 13yrs; weekly training volume 10.4 ± 4.1 h/week; handgrip maximum voluntary contraction (MVC) 46.3 ± 11.5 kg] divided into 2 matched groups (24 healthy MA and 24 MA with morbidity) and 10 healthy middle-aged non-athlete controls (age 48.3 ± 8.3 years; MVC 40.4 ± 14.8 kg) performed 5 min of forearm occlusion including 1 min handgrip isometric contraction (40%MVC) followed by 5 min recovery. Continuous beat-by-beat BP was recorded using finger plethysmography. Age-graded performance (AGP) was calculated to compare race performances among MA. Healthy Master cyclists were further grouped into middle-age (age 46.2 ± 6.4 years; N:12) and old-age (age 65.0 ± 7.7 years; N:12) for comparison with middle-aged non-athlete controls.

**Results:**

Healthy and morbidity MA groups showed similar BP responses during forearm occlusion and AGP (90.1 ± 4.3% and 91.0 ± 5.3%, *p *> 0.05, respectively). Healthy and morbidity MA showed modest correlation between the BP rising slope for 40%MVC ischaemic exercise and AGP (*r* = 0.5, *p *< 0.05). MA showed accelerated SBP recovery after cessation of ischaemic handgrip exercise compared to healthy non-athlete controls.

**Conclusion:**

Our findings associate long-term athletic training with improved BP recovery following ischaemic exercise regardless of age or reported morbidity. Exaggerated BP in Master cyclists during ischaemic exercise was associated with lower AGP during the World Master Cycling Championships.

**Supplementary Information:**

The online version contains supplementary material available at 10.1007/s00421-021-04828-9.

## Introduction

Elevated exercise blood pressure (BP) is a prevalent risk factor for cardiovascular (CV) diseases in sedentary individuals (Schultz et al. 2012) and elite athletes (Berge et al. 2015). Regular moderate exercise can maintain healthy vascular function (Maron et al. 2001; Montero et al. 2015; Pollock et al. 2018), and reduce the risk of hypertension (Fagard 2001; Mora et al. 2007). However, there is a controversy about the impact of lengthy and intense training on cardiovascular health and the incidence of hypertension (Kujala et al. 1999; Kim et al. 2012; Andersen et al. 2013, 2020; Schwartz et al. 2014; Eijsvogels and Maessen 2017). Master athletes provide an opportunity to study vascular function and BP responses of exceptionally active individuals, since they typically train regularly and compete in athletic events at very high intensity (Tanaka et al. 2019). Middle-aged and elderly master athletes show improved vascular function with optimized blood flow and BP regulation at rest and during exercise (Montero et al. 2015). However, some vulnerable master athletes are at risk of developing adverse cardiovascular morbidity (Andersen et al. 2013). Indeed, a prevalence of 10% with established respiratory and cardiovascular diseases was found in a master athletes’ cohort (Shapero et al. 2016). These morbidities are often connected to an impaired BP regulation during exercise despite a normotensive status at rest (Currie et al. 2017), indicating an early state of hypertensive disease.

The increase in BP during exercise is regulated through a feed-forward central command mechanism (Goodwin et al. 1972) and affected by the baroreflex (Bristow et al. 1971), and the exercise pressor reflex (EPR) (McCloskey and Mitchell 1972) feedback mechanisms. Activation of muscle afferents (III–IV) by mechanical and metabolic stimuli evoke EPR which increases sympathetic outflow to the heart and resistance vessels (Seals et al. 1988; Rowell and O'Leary 1990; Amann et al. 2011; Sidhu et al. 2015). The magnitude of EPR during fatiguing isometric exercise increases with aging and cardiovascular morbidity (Petrofsky and Lind 1975; Delaney et al. 2010). Endurance training showed blunted EPR (Somers et al. 1992; Mostoufi-Moab et al. 1998) and long-term endurance athletes exhibit lower EPR than untrained individuals (Kölegård et al. 2013). Nevertheless, susceptible master athletes are at risk of an exaggerated BP response during exercise (Pressler et al. 2018). An elevated BP during a progressive maximal cardiopulmonary exercise test (CPET) was associated with a decreased exercise capacity in elite young athletes with autonomic dysfunction (Mazic et al. 2015). However, the connection of an elevated exercise BP with sport performance is not well documented.

Forearm occlusion, ischaemic isometric exercise and post-exercise circulatory occlusion have shown to elevate the muscle III/IV afferent feedback and exaggerate the BP response (Papelier et al. 1997; Fisher et al. 2007; Faisal et al. 2010). These interventions induce minimum changes in muscle metabolism (i.e. O_2_ stores and phosphocreatine) during occlusion and fully return to base line state with 5 min of recovery (Hampson and Piantadosi 1988; Blei et al. 1993; Boushel et al. 1998). Therefore, the aim of the present study was to examine the BP response to a fatiguing occlusion protocol in Master cyclists presenting with- and without morbidities and its impact on their sport performance during World Master Track Cycling Championships. Our hypothesis was that Master athletes with morbidity would experience an exaggerated EPR and BP response to the fatiguing occlusion protocol compared to Master athletes without morbidity, and that these athletes would have a lower performance during the competition.

## Methods

### Participants

Forty-eight non-smokers master athletes (F:13) competing at the 2019 World Master Track Cycling Championships in Manchester (UK) were recruited to participate in this study along with a healthy non-athletic control group (N:10). All participants completed a general health questionnaire and provided detailed medical history and use of medications. MA were divided into two matched groups (N:24), healthy MA or MA with reported morbidity based on the presence or history of cardiovascular diseases (N:9), respiratory diseases (N:4) or any other pathology (N:11) that may alter BP at rest and during exercise (Souza et al. 2015; Christiansen et al. 2016; Sumner et al. 2016; Berta et al. 2019; Leeman and Kestelyn 2019). Reported comorbidities included hypertension, hypotension, blood clot, iliac arterial occlusive disease, thrombosis, myocarditis and cardiac arrythmias (atrial fibrillation), asthma, hypothyroidisms, history of breast, skin and prostate cancer, cox-arthritis, glaucoma, post-traumatic syndrome disorder (details of medications are shown in Table E1—Online Supplement). The healthy and morbidity MA groups were matched for age, sex, self-reported weekly training volume over the last year of training before competition (average: 11 h/week), and handgrip maximum voluntary contraction (MVC) (48.3 ± 10.3; 44.2 ± 12 kg, *p *> 0.05, respectively). Subsequently, the healthy MA group was divided into sub-groups of middle-aged MA (48.9 ± 9.2 years, N:12) and older MA (65.8 ± 10.2 years, N:12) and were compared with a healthy middle-aged non-athlete controls (48.3 ± 8.3 years, N:10). This study was approved by Manchester Metropolitan University Ethics committee (Approval ID: 11704). Participants provided written, informed consent and were requested to avoid caffeine intake for 12 h prior to participation in the study.

**Table 1:** Subject characteristics in healthy and morbidity Master athletes

Group	Healthy MA (24)	Morbidity MA (24)
Age (y)	57.2 ± 12.6	60.7 ± 12.5
Height (cm)	172.5 ± 6.9	169.4 ± 6.3
Weight (kg)	75.7 ± 11.3	76.8 ± 12.7
BMI (kg/m^2^)	25.3 ± 2.7	25.3 ± 3.4
Training (h/week)	11.1 ± 4.6	9.8 ± 3.3
MVC 40% (kg)	19.5 ± 4.3	17.7 ± 4.8
Resting SBP (mmHg)	131.3 ± 12.6	133.1 ± 12.7
Resting MAP (mmHg)	94.7 ± 7.8	94.4 ± 7.8
Resting DBP (mmHg)	77.1 ± 7.3	77.5 ± 7.7

Values are means ± SD

*BMI* body mass index, *DBP* diastolic blood pressure, *MAP* mean arterial pressure, *MVC* maximal voluntary contraction, *SBP* systolic blood pressure

### Experimental design and procedures

**Fig. 1:**
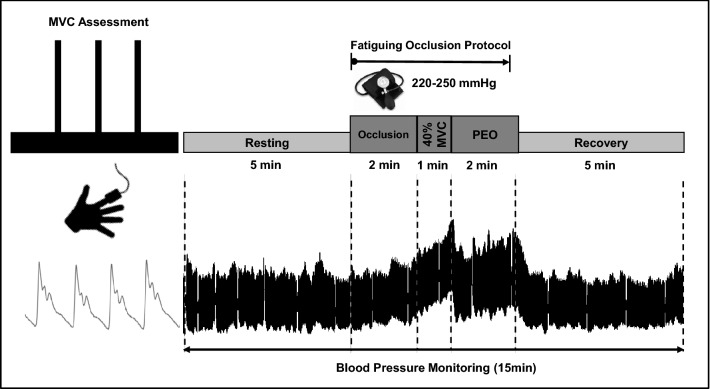
Experimental testing protocol on top and a representative recordings of blood pressure using finger plethysmography at the bottom. *MVC* maximal voluntary contraction, *PEO* post-exercise occlusion

All participants completed a fatiguing occlusion protocol in one session at least 24 h prior to or following participation in the cycling competition. Following 15 min of comfortable upright sitting, participants performed three handgrip maximal voluntary contractions (MVC) with a 1-min recovery period between contractions. MVC was assessed with a grip force transducer attached to data acquisition system (Power lab—ADI Instruments Systems, Oxford, UK) and calculated as the average of 1-s peak strength in the best two trials. The fatiguing occlusion protocol was adapted from previous studies on BP response and metabo-receptors activity to ischemia (Fisher and White 1999; Faisal et al. 2010; Delaney et al. 2010; Currie et al. 2017). It included 5 min of resting, 5 min of forearm occlusion including 1 min of isometric contraction at 40% MVC and 5 min recovery (Fig. 1). Participants sat in an upright position using the dominant arm extended at the heart level. Circulatory occlusion of the brachial artery distal to the elbow was achieved by rapid inflation of a standard blood pressure cuff to 220–250 mmHg (Elite BFR Occlusion Cuffs, © The Occlusion Cuff). Exercise ischemia was assessed by the absence of the radial artery pulse during the occlusion protocol (Crenshaw et al. 1988; Patterson et al. 2019), and the occlusion period was terminated by rapid deflation of the occlusion cuff. Beat-by-beat BP was measured throughout the testing protocol using finger plethysmography (Human NIBP nano, ADI Instruments Systems, Oxford, UK). Finger plethysmography has been shown to be a valid and reliable tool for assessing blood pressure during hemodynamic changes (Waldron et al. 2017). Participants were asked to rate the intensity of pain discomfort using a 0–10 Numeric Rating Scale (Haefeli and Elfering 2006), throughout the 5 min fatiguing occlusion protocol (at 2, 3 and 5 min). The fatiguing occlusion protocol was completed in full by all participants, except for two participants who both reported light-headedness and nausea impairing their ability to complete the contraction, so their data were excluded from further analysis.

### Data analysis

Systolic blood pressure (SBP), mean arterial pressure (MAP) and diastolic blood pressure (DBP) values were calculated as a mean of each phase of the fatiguing occlusion protocol [occlusion, ischaemic exercise (MVC40% during occlusion), post-exercise occlusion (PEO)], and at the last min of resting and recovery periods before and after the fatiguing occlusion protocol (Fig. 1). Delta BP among phases of the testing protocol were calculated as the differences between BP means. Beat-by-beat SBP and MAP dynamics were assessed by slope analysis throughout ischaemic isometric exercise, PEO phases and during the first 2 min of recovery after cuff release. Age-graded performance (AGP) analysis was used to calculate the race performances of the MA cohort with respect of their specific age groups and race events, expressed as a percentage of the corresponding age-group world records for that particular race event (Bird et al. 2003). Some Master athletes were competing in different events (200–500 m and/or 2000 m), and in these cases, AGP was recorded for the individual athlete as the highest AGP from any of the race events they competed in. Official race results were obtained from the 2019 World Master Track Cycling Championships website.

### Statistical analysis

All variables met the parametric assumption for normality of distribution and the homogeneity of variance using Shapiro–Wilk and Leaven’s tests. A sample size of 24 was estimated to provide 80% power to detect differences in BP responses between Master cyclists with or without morbidity, based on a SD of one unit, α of 0.05, and a two-tailed test of significance. An unpaired *T* test was performed for all the participants’ characteristics, pain, AGP, BP slopes between healthy and morbidity MA groups. A one-way repeated measure ANOVA with Bonferroni post hoc analysis was performed to examine differences in mean and delta SBP, MAP, DBP responses at different phases of the testing protocol between healthy MA and morbidity MA, as well as between old and middle-aged MA, and healthy middle-aged controls. A simple linear regression analysis was performed on the MA cohort to examine correlation between the raising slope of SBP and MAP during ischaemic isometric handgrip contraction and AGP. All the statistical analysis were performed using the GraphPad Prism 8 statistical analysis software. Data are reported as mean ± SD with statistically significance accepted at *p *< 0.05.

## Results

### Morbidity effect in Master athletes

The healthy and morbidity MA groups were of similar age, height, weight, MVC, and weekly training volume (Table 1). Both groups showed similar changes in BP responses (mean, delta and slope) (Fig. 1, Tables E2, E3—Online Supplement) and reported similar pain discomfort during the three phases of the fatiguing occlusion protocol (Table E4—Online Supplement). Within the morbidity MA group, there were no differences in BP responses between MA with cardiovascular diseases and MA with other reported conditions (Fig. 1E—Online Supplement). There were no significant differences in AGP between healthy and morbidity MA groups (90.13 ± 4.26 and 90.98 ± 5.33%, *p *> 0.05).

An inverse correlation was found between the slope of increased SBP and MAP during ischaemic exercise and AGP (r = 0.50, r = 0.46, *p *< 0.05 for both- Fig. 3a, b).

### Aging and training effect in healthy Master athletes

Based on the inclusion criteria of the healthy non-athletes control group, there was a significant difference in age with older MA and significant difference in weekly training volume with both middle-aged and older MA (Table 2). Old and middle-aged MA showed similar changes in SBP, MAP, DBP at rest, occlusion, ischaemic exercise, PEO and recovery phases (*p *> 0.05 for all, Fig. 4; Table E5, E6—Online Supplement). There were no differences between MA groups and middle-aged healthy controls in the rise of slope of SBP or MAP during ischaemic exercise. However, compared to the healthy middle-age group, both old and middle-age MA groups showed a steeper decrease in SBP and MAP slopes over 1 and 2 min of PEO (i.e. SBP 1 min: 0.38 ± 0.23, 0.35 ± 0.20 vs. 0.15 ± 0.10, *p *< 0.05 for both) with larger delta (Table E6—Online Supplement). There was a faster BP recovery from the fatiguing occlusion protocol in the MA groups with a steeper decrease in SBP during 1 and 2 min of recovery compared to the healthy middle-aged controls (*p *< 0.05, Fig. 4; Table E6—Online Supplement). Moreover, the middle-aged healthy MA group showed a lower SBP at the end of recovery phase compared to the middle-aged healthy controls (132.3 ± 8.2 vs. 143.0 ± 14.1 mmHg, *p *< 0.05, Fig. 4; Table E5—Online Supplement). All groups reported similar pain discomfort during the three phases of the fatiguing occlusion protocol (Table E6 E7—Online Supplement).

**Table 2:** Subject characteristics in middle-aged and older MA and middle-aged non-athlete controls

Group	Middle-aged non-athlete controls (10)	Middle-aged master athletes (12)	Older master athletes (12)
Age (y)	48.3 ± 8.3	48.9 ± 9.1	65.8 ± 10.2*
Height (cm)	172.4 ± 6.4	175.5 ± 7.4	169.8 ± 6.1
Weight (kg)	77.2 ± 9.6	77.4 ± 11.3	70.2 ± 11.2
BMI (kg/m^2^)	25.9 ± 3.0	25.0 ± 2.3	23.8 ± 3.2
Training (h/week)	3.7 ± 1.5*	10.5 ± 2.8	11.6 ± 6.2
MVC (kg)	40.4 ± 14.8	49.7 ± 11.3	46.9 ± 10.9
Resting SBP (mmHg)	136.9 ± 11.6	129.4 ± 8.4	133.2 ± 16.4
Resting MAP (mmHg)	98.6 ± 11.59	94.9 ± 6.4	94.5 ± 9.7
Resting DBP (mmHg)	79.4 ± 11.9	77.5 ± 6.1	76.6 ± 8.8

Values are means ± SD

*BMI* body mass index, *DBP* diastolic blood pressure, *MAP* mean arterial pressure, *MVC* maximal voluntary contraction, *SBP* systolic blood pressure

*p < 0.05 old MA vs. middle-age MA vs and none-athlete controls

## Discussion

To the best of our knowledge, this is the first study of exercise-related BP dynamics in MA with and without morbidity during an international competition. The main findings of this study are as follows: 1) In contrast to our hypothesis, MA with underlying morbidity showed similar BP responses at rest, occlusion, ischaemic exercise, post-ischaemic exercise and recovery phases as healthy MA, 2) the slope of SBP rise during ischaemic isometric exercise was inversely correlated with AGP during the cyclist competition, and 3) older MA showed similar BP responses compared to healthy middle-age non-athletes and both older and middle-aged MA showed a faster BP recovery during PEO and following the fatiguing occlusion protocol compared to middle-aged healthy controls.

### Impact of underlying morbidity on BP regulation in MA

Our findings associate athletic training with improved BP regulation under occlusion, ischaemic exercise and during recovery after exercise occlusion, regardless of underlying morbidity in our MA participants. The link between altered BP responses to exercise and underlying morbidity has been studied in detail amongst the general population (Mundal et al. 1994; Kjeldsen et al. 2001) and patient groups (Schultz et al. 2012; Grotle and Stone 2019; Downey et al. 2017; Delaney et al. 2010; Piepoli and Coats 2007), but less is known about BP responses for athletic populations (Andersen et al. 2020). Master athletes are characterised by very high levels of exercise training and they typically present with fewer underlying health conditions compared with non-athletic individuals of the same age (Tanaka et al. 2019). However, long-term strenuous exercise could potentially lead to cardiovascular dysfunction (O'Keefe et al. 2012; Stergiou and Duncan 2018) and masked hypertension with altered cardiac function being a highlighted risk among master athletes (Trachsel et al. 2015).

The increases of blood pressure during isometric exercise are linked to the activation of peripheral mechano- and metabo-receptors (groups III and IV afferents) in musculature which activate central sympathetic outflow driving generalised arterial constriction (Goodwin et al. 1972; McCloskey and Mitchell 1972; Kaufman et al. 1983). Cardiovascular diseases are characterized by altered functionality of peripheral or central influences on sympathetic outflow, which is associated with exaggerated increases of BP and vascular resistance during exercise (Murphy et al. 2011; Mitchell 2017; Aoki et al. 1983). Overactivation of sympathetic outflow and pressor responses during post-exercise ischemia, mediated by the muscle metaboreflex, was linked to an increased BP in hypertensive individuals (Delaney et al. 2010). However, Currie et al. reported similar arterial stiffness and sympathetic reactivity during isometric handgrip exercise and post-exercise muscle ischemia in healthy endurance MA with and without exaggerated BP to graded dynamic exercise (Currie et al. 2017).

**Fig. 2:**
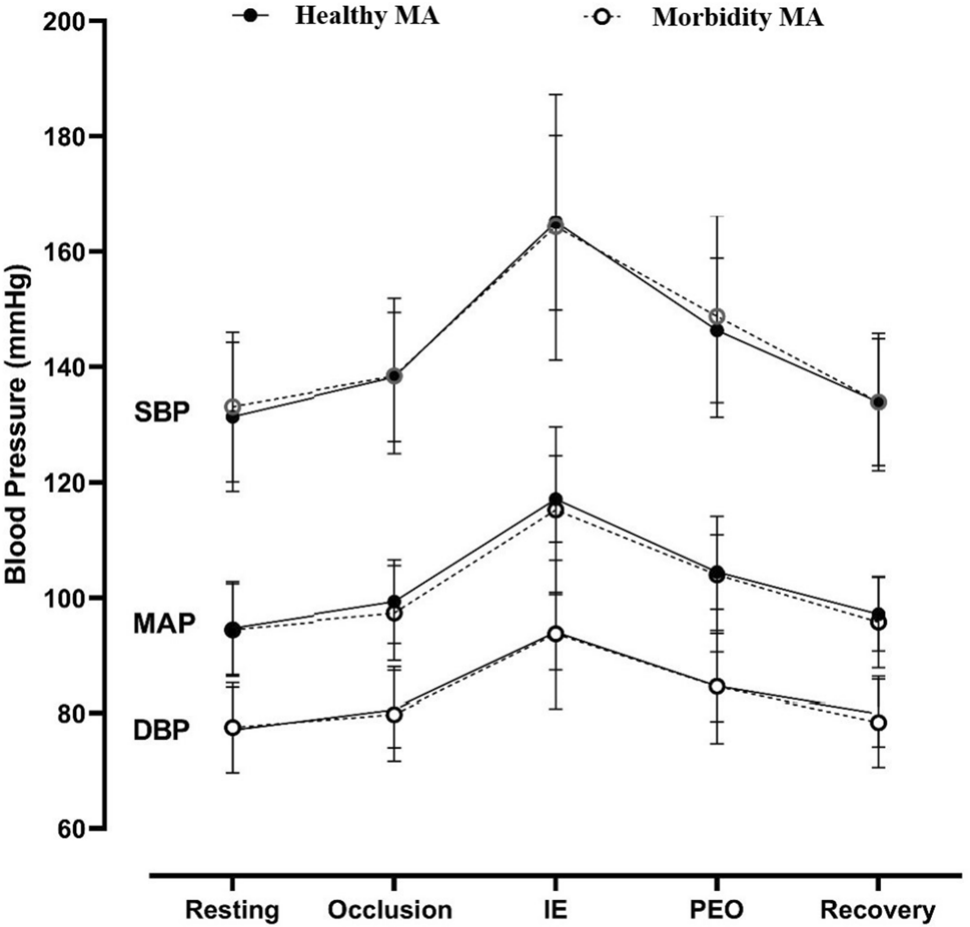
Blood Pressure responses in healthy and Master cyclists with morbidity groups at resting, occlusion, ischaemic exercise (IE), post-exercise occlusion (PEO), and recovery. Values are means ± SE

A proportion of the master athletes in our study reported underlying medical conditions, albeit controlled and without any symptoms that interrupted their ability to compete in world championship events. Despite the presence of morbidity, we did not find differences in BP responses during occlusion, IE or during post-exercise cessation compared to master cyclists free from morbidity (Fig. 2). Therefore, there may be a possible protective effect of long-term intense exercise on BP regulation even in the presence of morbidity within elite master athletes.

**Fig. 3:**
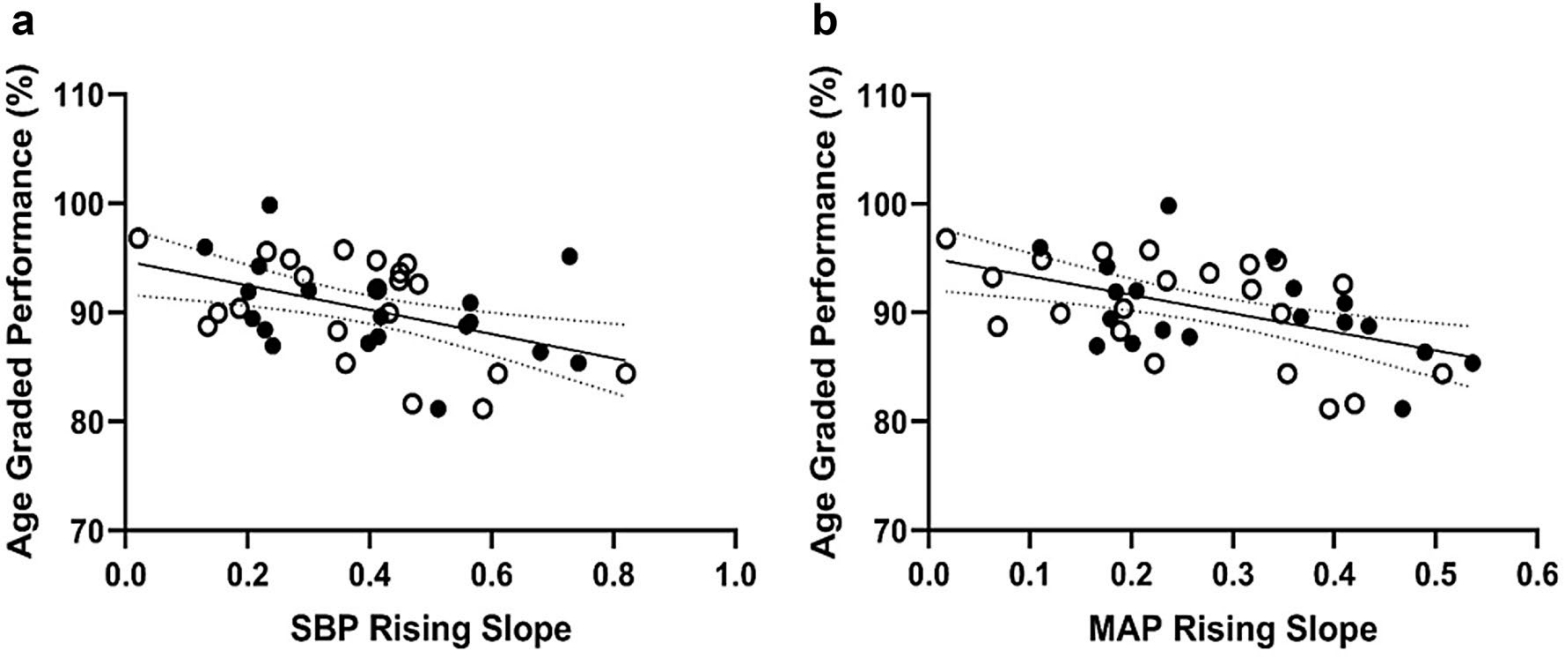
Linear regression of age-graded performance and the slopes of SBP rises (**a**) and MAP rises slope (**b**) during ischaemic handgrip isometric exercise in Master athletes cohort [healthy (solid circles) and Master athletes with morbidity (open circles)]

While an exaggerated exercise BP in MA is considered a compensatory mechanism to maintain adequate perfusion for active muscles (Currie et al. 2017), increased BP during dynamic exercise was associated with lower exercise capacity in hypertensive patients (Fagard et al. 1988; Pickering 1987; Lim et al.^1996^) and elite young athletes (Mazic et al. 2015). Among over 200,000 recreational to elite skiers, including 8% with morbidity, higher performance in a Nordic skiing race at the Vasaloppet Swedish competition was strongly associated with a lower incidence of hypertension (Andersen et al. 2020). In MA (cyclists), we found an inverse correlation between the rise in BP during ischaemic isometric exercise and AGP regardless of the underlying morbidity in our MA groups (Fig. 3). In approximately 10% with hypertensive resting BP of our MA population, exaggerated blood BP responses during IE (above 190 and 210 mmHg for women and men, respectively) was associated with lower AGP in the world cycling competition.

### Impact of age and training on BP regulation

**Fig. 4:**
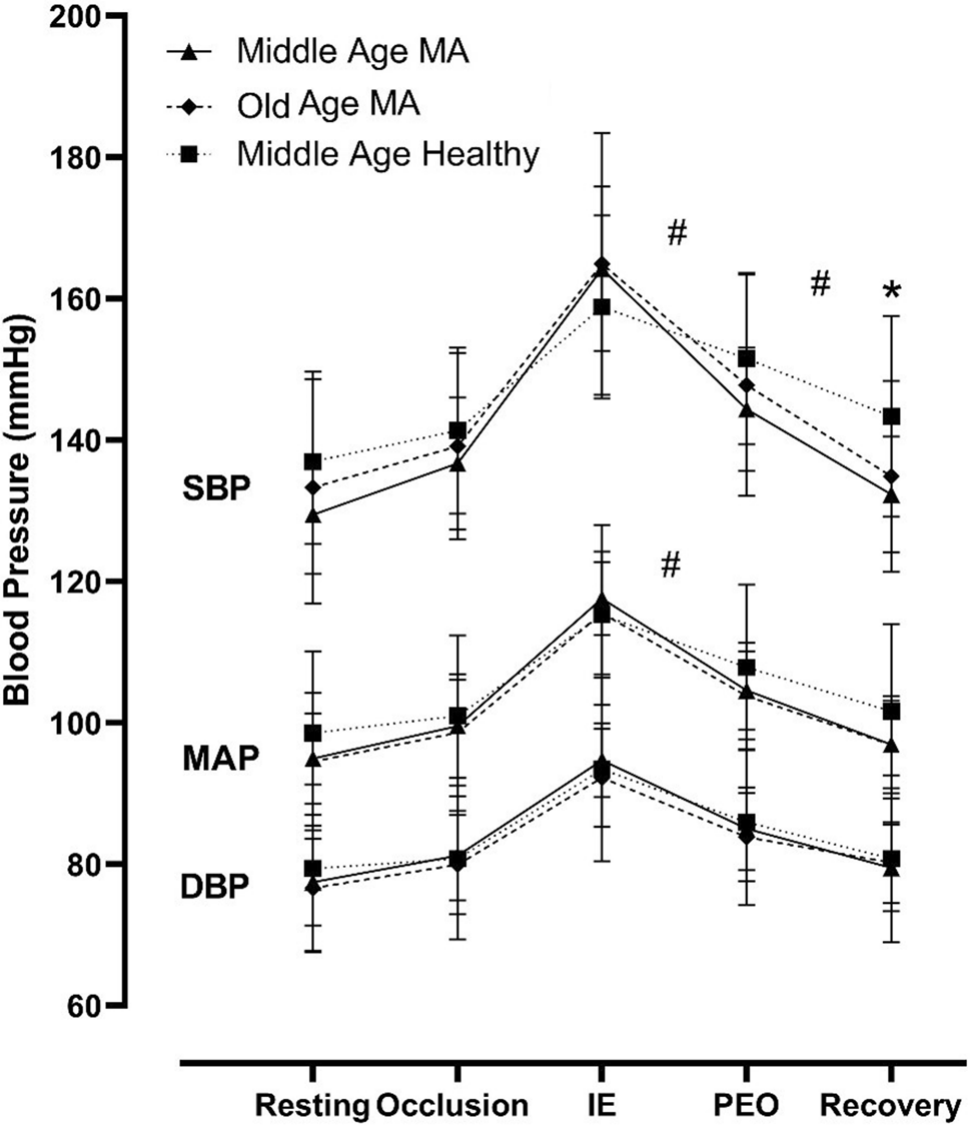
Blood Pressure responses in healthy middle-aged and older Master cyclists’ groups and healthy middle-aged non-athletic controls at rest, occlusion, ischaemic exercise (IE), post-exercise occlusion (PEO), and recovery. Values are means ± SE. *p < 0.05 for Middle-age MA vs. non-athlete controls; #*p* < 0.05 for middle-age and old MA vs. non-athlete controls in SBP and MAP slopes

Age-associated changes in vascular structure and loss of central arteries elasticity (Kelly et al. 1989; Vaitkevicius et al. 1993; Franklin et al. 1997; McEniery et al. 2005), and chronic elevation of muscle sympathetic nerve activity (MSNA) (Ng et al. 1993; Studinger et al. 2009) are key determinants for impaired BP responses in sedentary aging. In contrast, endurance master athletes (cyclists, runners and swimmers, triathletes) show improved vascular function compared to sedentary healthy age-matched controls (DeVan and Seals 2012). This includes increased conduit artery cross-sectional area (Montero et al. 2015) higher arterial compliance (Tanaka et al. 2000; Monahan et al. 2001; Nualnim et al. 2011), less hypertrophy of the arterial wall (DeVan and Seals 2012), lower arterial stiffness (Vaitkevicius et al. 1993; Tanaka et al. 1998), enhanced endothelial function and improved blood flow circulation (Montero et al. 2015) to optimize blood flow and BP regulation at rest and during exercise (Montero et al. 2015). Exaggerated BP responses (EBPR) with ageing were reported during dynamic exercise (Fisher et al. 2010a, 2007), but still controversial during isometric exercise (Houssiere et al. 2006; Lalande et al. 2014; Sidhu et al. 2015). Our results showed that BP responses were comparable in older MA, middle-aged MA and middle-aged healthy non-athletic controls during occlusion and IE (Fig. 4). It can be assumed that the stimulus of the mechano- or metabo-receptors was similar for all groups. This may decrease the ageing effect on the contribution of muscle III/IV afferents to stimulate BP under ischaemic or isometric exercise conditions (Sidhu et al. 2015). The impact of lifetime training on vascular sympathetic activity in master athletes remains unclear and showed inconsistent findings of higher (Ng et al. 1993; Wakeham et al. 2019) or no changes (Studinger et al. 2009) in resting MSNA compared to healthy untrained individuals. However, it is noteworthy that increased BP during occlusion or ischaemic exercise does not necessarily follow elevated vascular sympathetic activity at rest (Taylor and Tan 2014).

The accelerated decrease of BP during PEO in middle-aged and older MA compared to middle-aged healthy non-athletic controls (Fig. 4) may suggest robust reactivation of cardiac parasympathetic tone and increased cardiac baroreflex sensitivity in our MAs following the inhibition of central command and removal of mechano-reflex stimulation (O’Leary, 1993; Carrington and White 2001; Fisher et al. 2010b), causing a rapid reduction of BP despite sustained high MSNA due to muscle metaboreflex activation (Fadel 2015; Mark et al. 1985; Ichinose et al. 2006; Ng et al. 1994). Moreover, the accelerated recovery of BP in the master cyclists post-fatiguing occlusion protocol could be related to enhanced vasodilatory capacity (Ferguson and Brown 1997; Wakeham et al. 2019) and/or increased cardiovagal baroreflex sensitivity (Monahan et al. 2000), that led to a rapid decrease of BP. However, further and detailed measurement should be implemented in the future to clarify the contribution of chronic training on the interaction between muscle metaboreflex with arterial and cardiac baroreflex to the neural control of BP during PEO and recovery in MA populations.

## Study limitations

A possible limitation of this study was that the data from Master cyclists were collected during World Championships, which may have elevated BP responses due to anticipation, excitement, dehydration, or prior exercise. However, steps were taken to avoid strenuous exercise prior to testing and to ensure hydration before testing. Additionally, there were no differences in baseline BP in Master cyclists compared to middle-aged healthy non-athletic controls who did the measurements in a quiet laboratory setting. Our main finding of similar BP responses between healthy MA and those with underlying morbidity is likely to be influenced by medication. The morbidity MA group has been screened and their medications were eligible to be used during international events (Maron et al. 2001; Van Hare et al. 2015). More invasive techniques of microneurography were not possible due to testing constraints, but these would have provided greater insights into the possible mechanisms influencing BP responses during IE, PEO and recovery. In addition, only 25% of the MA were female due to lower participation of females in the events compared to male athletes. More female athletes should be studied in the future as endurance training showed only higher resting MSNA mainly in women MA (Ng et al. 1993).

## Conclusion

These findings of accelerated blood pressure recovery after cessation of ischaemic hand grip exercise in MA associate long-term athletic training with improved blood pressure dynamics regardless of age or underlying morbidity. Middle-aged and older MA showed accelerated BP recovery after cessation of ischaemic hand grip exercise compared with non-athletic middle-aged adults. This response was similar between MA with and without underlying morbidity. Exaggerated BP in MA during ischaemic exercise was associated with lower AGP during the World Master Track Cycling Championships.

## Supplementary Information

Below is the link to the electronic supplementary material.

Supplementary file1 (DOCX 320 KB)

## Data Availability

Raw data are available upon request.

## Abbreviations

AGP: Age-Graded Performance
ANOVA: Analysis of Variance
BP: Blood Pressure
BMI: Body Mass Index
CPET: Cardiopulmonary Exercise Test
DBP: Diastolic Blood Pressure
EBPR: Exaggerated Blood Pressure Response
EPR: Exercise Pressor Reflex
HG: Handgrip
IE: Ischaemic Exercise
MAP: Mean Arterial Pressure
MA: Master Athletes
MSNA: Muscle Sympathetic Nerve Activity
MVC: Maximal Voluntary Contraction
PEO: Post-Exercise Occlusion
SBP: Systolic Blood Pressure
