# Supplementary material for: The association of elevated blood pressure during ischaemic exercise with sport performance in Master athletes with and without morbidity
Source: Eur J Appl Physiol. 2021 Oct 15;122(1):211–21. doi: 10.1007/s00421-021-04828-9 (PMC8748359; doi:10.1007/s00421-021-04828-9)
Supplement: Supplementary file 1 — Supplementary file1 (DOCX 320 KB) [file 421_2021_4828_MOESM1_ESM.docx]

Online Supplement

**The association of elevated blood pressure during ischaemic exercise with sport performance in Master athletes with and without morbidity**

**Fabio Zambolin^1^, Jamie S. McPhee^1,2^, Pablo Duro Ocaña^3^, Bergita Ganse^4^, Liam Bagley^2,3^, Azmy Faisal^1,2,5^**

^1^Department of Sport and Exercise Sciences, Musculoskeletal Science and Sports Medicine Research Centre, Faculty of Science & Engineering, Manchester Metropolitan University, Manchester, UK.

^2^Manchester Metropolitan University Institute of Sport, Manchester, UK.

^3^Department of Life Sciences, Musculoskeletal Science and Sports Medicine Research Centre, Faculty of Science & Engineering, Manchester Metropolitan University, Manchester, UK.

^4^Saarland University Hospital, Innovative Implant Development, Homburg, Germany

^5^Faculty of Physical Education for Men, Alexandria University, Alexandria, Egypt

**Corresponding Author:** Dr. Azmy Faisal

Department of Sport and Exercise Sciences, Manchester Metropolitan University, All Saints Building, Manchester, UK, M15 6BH; Tel: +44 (0) 1612475627; e-mail: azmy.faisal@mmu.ac.uk

**ORCID ID**: A.F. (0000-0001-5019-7292)

**Running Head:** *Blood Pressure Regulation in Master Athletes*


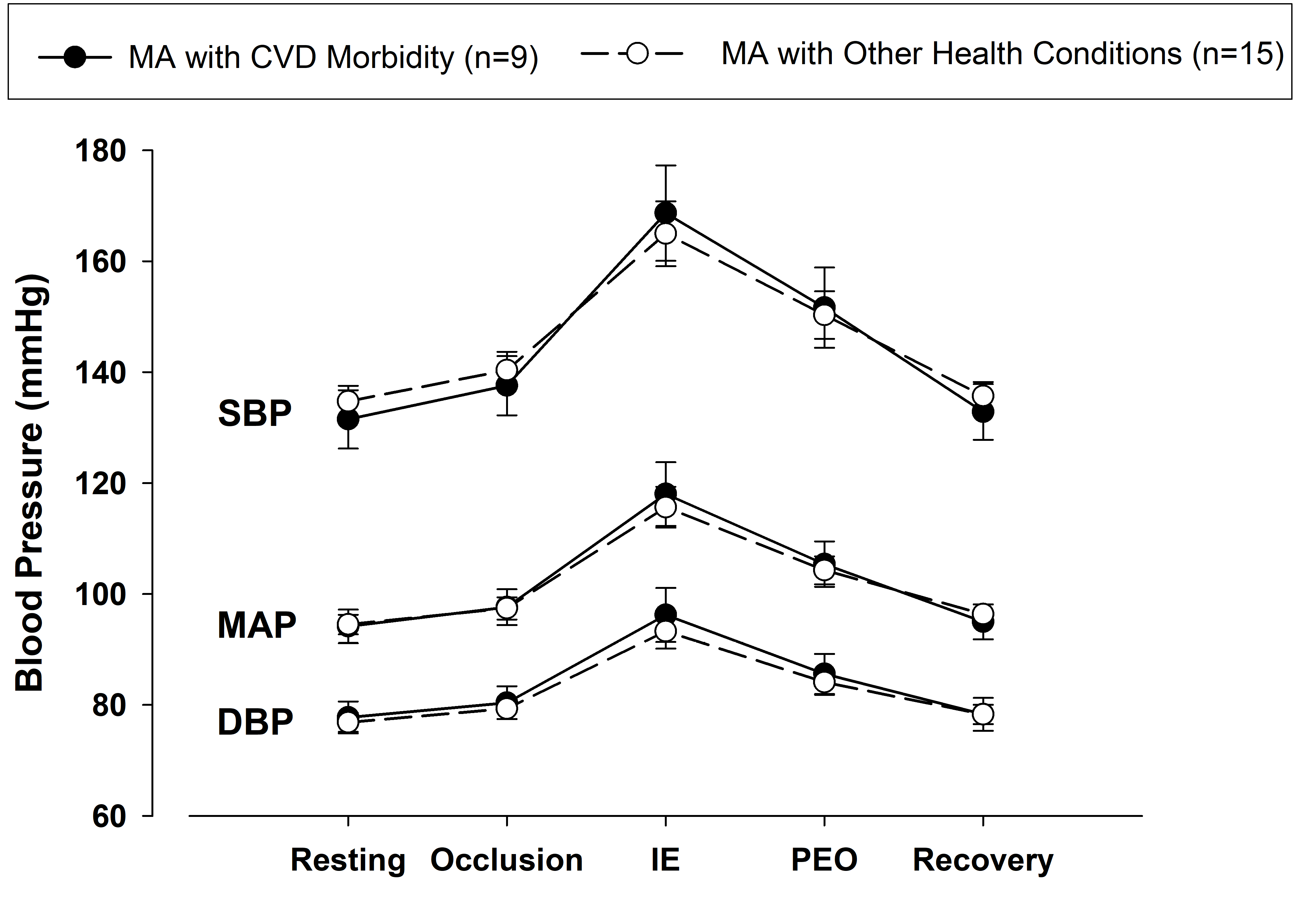


**Fig. E1.** Blood pressure responses in Master athletes with cardiovascular morbidity and Master athletes with other reported health conditions at resting, occlusion, ischaemic exercise (IE), post exercise occlusion (PEO), and recovery.

Values are means ± SE.

| Table E1. List of Medications | |
| --- | --- |
| Medication | **Number of Patients** |
| Anti hypertensive | 1 |
| Anti-GERD | 3 |
| Statins | 2 |
| Aspirin | 2 |
| Thyroid replacement therapy | 3 |
| SABA | 4 |
| ICS | 1 |

*GERD, gastroesophageal reflux disease; SABA, short-acting β2 agonist; ICS, inhaled corticosteroids.*

| Table E2. Blood Pressure Responses in Healthy and Morbidity Master Athletes Groups | | |
| --- | --- | --- |
| Group | **Healthy MA (24)** | **Morbidity MA (24)** |
| SBP Resting (mmHg) | 131.3 ± 12.6 | 133.1 ± 12.7 |
| SBP Occlusion (mmHg) | 137.9 ± 11.0 | 138.1 ± 13.2 |
| SBP Ischemic Exercise (mmHg) | 164.8 ± 15.0 | 164.1 ± 22.7 |
| SBP Post Exercise Occlusion (mmHg) | 146.0 ± 12.2 | 148.5 ± 17.1 |
| SBP Recovery (mmHg) | 133.6 ± 10.8 | 133.6 ± 11.7 |
| MAP Resting (mmHg) | 94.7 ± 7.9 | 94.4 ± 7.9 |
| MAP Occlusion (mmHg) | 99.1 ± 7.1 | 97.1 ± 8.0 |
| MAP Ischemic Exercise (mmHg) | 116.8 ± 7.3 | 114.9 ± 14.0 |
| MAP Post Exercise Occlusion (mmHg) | 104.2 ± 6.3 | 103.7 ± 9.9 |
| MAP Recovery (mmHg) | 96.9 ± 6.3 | 95.5 ± 7.7 |
| DBP Resting (mmHg) | 77.1 ± 7.3 | 77.5 ± 7.7 |
| DBP Occlusion (mmHg) | 80.6 ± 6.4 | 79.7 ± 7.9 |
| DBP Ischemic Exercise (mmHg) | 93.8 ± 6.4 | 93.4 ± 12.5 |
| DBP Post Exercise Occlusion (mmHg) | 84.4 ± 5.8 | 84.4 ± 9.5 |
| DBP Recovery (mmHg) | 79.9 ± 5.7 | 78.3 ± 7.7 |
| SBP Delta Rest/Occl (mmhg) | 6.5 ± 3.9 | 5.1 ± 6.1 |
| SBP Delta Occl/IE (mmHg) | 26.9 ± 9.5 | 25.9 ± 12.3 |
| SBP Delta IE/PEO (mmHg) | 18.8 ± 10.9 | 15.6 ± 9.8 |
| SBP Delta PEO/Recovery (mmHg) | 12.5 ± 6.3 | 14.9 ± 10.9 |
| MAP Delta Rest/Occl (mmhg) | 4.3 ± 2.8 | 2.6 ± 3.1 |
| MAP Delta Occl/IE (mmHg) | 17.8 ± 5.3 | 17.9 ± 8.6 |
| MAP Delta IE/PEO (mmHg) | 12.6 ± 5.6 | 11.3 ± 6.3 |
| MAP Delta PEO/Recovery (mmHg) | 7.3 ± 3.6 | 8.2 ± 6.5 |
| DBP Delta Rest/Occl (mmhg) | 3.5 ± 2.7 | 2.2 ± 2.4 |
| DBP Delta Occl/IE (mmHg) | 13.2 ± 4.3 | 13.7 ± 7.2 |
| DBP Delta IE/PEO (mmHg) | 9.3 ± 4.1 | 9.1 ± 5.6 |
| DBP Delta PEO/Recovery (mmHg) | 4.6 ± 3.0 | 6.0 ± 4.8 |

*Values are means ± SD; groups did not differ significantly for any of the variables (p>0.05).*

| Table E3. Blood Pressure Slope Analysis in Healthy and Morbidities Master Athletes Groups | | |
| --- | --- | --- |
| Group | **Healthy MA (24)** | **Comorbidities MA (24)** |
| SBP Slope HG | 0.41 ± 0.18 | 0.39 ± 0.17 |
| SBP Slope 1 PEO | -0.37± 0.21 | -0.26 ± 0.17 |
| SBP Slope 2 PEO | -0.14 ± 0.09 | -0.10 ± 0.06 |
| SBP Slope 1 Recovery | -0.18 ± 0.09 | -0.15 ± 0.22 |
| SBP Slope 2 Recovery | -0.09 ± 0.07 | -0.09 ± 0.10 |
| MAP Slope HG | 0.31 ± 0.12 | 0.27 ± 0.13 |
| MAP Slope 1 PEO | -0.20 ± 0.15 | -0.16 ± 0.10 |
| MAP Slope 2 PEO | -0.04 ± 0.07 | -0.04 ± 0.06 |
| MAP Slope 1 Recovery | -0.07 ± 0.08 | -0.08 ± 0.13 |
| MAP Slope 2 Recovery | -0.02 ± 0.06 | -0.03 ± 0.06 |

*Values are means ± SD; groups did not differ significantly for any of the variables (p>0.05).*

| Table E4. Pain Responses in Healthy and Morbidity Master Athletes Groups | | |
| --- | --- | --- |
| Group | **Healthy MA (24)** | **Comorbidities MA (24)** |
| Pain – Discomfort Occlusion | 1.7 ± 1.3 | 1.7 ± 1.1 |
| Pain – Discomfort IE | 2.9± 2.0 | 3.6 ± 1.4 |
| Pain – Discomfort PEO | 4.7 ± 2.4 | 4.9 ± 1.6 |

*Values are means ± SD; groups did not differ significantly for any of the variables (p>0.05).*

| Table E5. Blood Pressure Responses in Middle-aged MA, Older MA and Middle-aged Non-athlete Controls | | | |
| --- | --- | --- | --- |
| Group | **Middle-aged Non-athlete Controls (10)** | **Middle-aged Master Athletes (12)** | **Older Master Athletes (12)** |
| SBP Resting (mmHg) | 136.9 ± 11.6 | 129.5 ± 8.4 | 133.3 ± 16.4 |
| SBP occlusion (mmHg) | 141.4 ± 11.8 | 136.7 ± 9.3 | 139.1 ± 13.2 |
| SBP Ischemic Exercise (mmHg) | 158.8 ± 13.0 | 164.2 ± 11.6 | 164.9 ± 18.5 |
| SBP Post Exercise Occlusion (mmHg) | 151.5 ± 12.1 | 144.4 ± 8.8 | 147.7 ± 15.7 |
| SBP Recovery (mmHg) | 143.0 ± 14.1* | 132.3 ± 8.2 | 134.8± 13.5 |
| MAP Resting (mmHg) | 98.6 ± 11.6 | 94.9 ± 6.4 | 94.5 ± 9.7 |
| MAP occlusion (mmHg) | 101.0 ± 11.4 | 99.5 ± 7.3 | 98.6 ± 7.5 |
| MAP Ischemic Exercise (mmHg) | 115.2 ± 12.7 | 117.6 ± 5.1 | 115.5 ± 8.7 |
| MAP Post Exercise Occlusion (mmHg) | 107.8 ± 11.7 | 104.6 ± 5.5 | 103.8 ± 7.5 |
| MAP Recovery (mmHg) | 101.6 ± 12.3 | 96.9 ± 6.2 | 96.9 ± 6.9 |
| DBP Resting (mmHg) | 79.4 ± 11.9 | 77.5 ± 6.1 | 76.6 ± 8.8 |
| DBP occlusion (mmHg) | 80.8 ± 11.4 | 81.2 ± 6.4 | 80.0 ± 7.0 |
| DBP Ischemic Exercise (mmHg) | 93.4 ± 13.0 | 94.7 ± 5.2 | 92.2 ± 6.9 |
| DBP Post Exercise Occlusion (mmHg) | 86.0 ± 11.7 | 85.0 ± 5.8 | 83.8 ± 6.2 |
| DBP Recovery (mmHg) | 80.8 ± 11.8 | 79.5 ± 6.2 | 80.2 ± 5.7 |
| SBP Delta Rest/Occl (mmhg) | 4.4 ± 3.9 | 7.3 ± 4.1 | 5.8 ± 4.0 |
| SBP Delta Occl/IE (mmHg) | 17.5 ± 3.4* | 27.5 ± 10.0 | 25.8 ± 8.9 |
| SBP Delta IE/PEO (mmHg) | 7.3 ± 3.5* | 19.9 ± 11.3 | 17.2 ± 10.0 |
| SBP Delta PEO/Recovery (mmHg) | 8.5 ± 5.2 | 12.1 ± 7.0 | 12.9 ± 6.0 |
| MAP Delta Rest/Occl (mmhg) | 2.4 ± 2.1 | 4.6 ± 2.8 | 4.1 ± 3.0 |
| MAP Delta Occl/IE (mmHg) | 14.3 ± 4.1 | 18.1 ± 6.4 | 16.9 ± 3.4 |
| MAP Delta IE/PEO (mmHg) | 7.4 ± 3.3* | 13.0 ± 5.8 | 11.7 ± 4.6 |
| MAP Delta PEO/Recovery (mmHg) | 6.2 ± 2.9 | 7.7 ± 3.9 | 6.9 ± 3.4 |
| DBP Delta Rest/Occl (mmhg) | 1.4 ± 1.4 | 3.7 ± 2.7 | 3.3 ± 2.9 |
| DBP Delta Occl/IE (mmHg) | 12.7 ± 4.7 | 13.5 ± 5.2 | 12.3 ± 3.3 |
| DBP Delta IE/PEO (mmHg) | 7.5 ± 3.6 | 8.7 ± 4.9 | 6.9 ± 4.1 |
| DBP Delta PEO/Recovery (mmHg) | 5.2 ± 2.3 | 5.5 ± 2.8 | 3.6 ± 3.0 |

*Values are means ± SD; *p <0.05 Middle-age MA vs Old MA vs Control.*

| Table E6. BP Slope Analysis in Middle-Aged and Older MA and Middle-Aged Non-Athlete Controls | | | |
| --- | --- | --- | --- |
| Group | **Middle-age Non-athlete Controls (10)** | **Middle-age Master Athletes (12)** | **Old Master Athletes (12)** |
| SBP Slope HG | 0.31 ± 0.09 | 0.39 ± 0.21 | 0.43 ± 0.17 |
| SBP Slope 1 PEO | -0.15 ± 0.10^#^ | -0.35 ± 0.20 | -0.38 ± 0.23 |
| SBP Slope 2 PEO | -0.05 ± 0.07^#^ | -0.14 ± 0.09 | -0.13 ± 0.10 |
| SBP Slope 1 Recovery | -0.06 ± 0.13^#^ | -0.17 ± 0.08 | -0.20 ± 0.11 |
| SBP Slope 2 Recovery | -0.01 ± 0.06^#^ | -0.09 ± 0.09 | -0.09 ± 0.05 |
| MAP Slope HG | 0.27 ± 0.12 | 0.28 ± 0.13 | 0.34 ± 0.12 |
| MAP Slope 1 PEO | -0.06 ± 0.04^#^ | -0.22 ± 0.13 | -0.21 ± 0.14 |
| MAP Slope 2 PEO | -0.02 ± 0.03^#^ | -0.06 ± 0.05 | -0.06 ± 0.05 |
| MAP Slope 1 Recovery | -0.06 ± 0.05 | -0.09 ± 0.04 | -0.09 ± 0.07 |
| MAP Slope 2 Recovery | -0.02 ± 0.01 | -0.04 ± 0.6 | -0.04 ± 0.02 |

*Values are means ± SD; # p <0.05 Middle-age MA vs Old MA vs Control.*

| Table E7 Pain Responses in Middle-Aged and Older MA and Middle-Aged Non-Athlete Controls | | | |
| --- | --- | --- | --- |
| Group | **Middle-aged Non-Athlete Controls (10)** | **Middle-aged Master Athletes (12)** | **Older Master Athletes (12)** |
| Pain – Discomfort Occlusion | 1.7 ± 1.6 | 1.6 ± 1.5 | 1.6 ± 1.3 |
| Pain – Discomfort IE | 2.0± 2.3 | 2.5 ± 2.1 | 2.5 ±2.7 |
| Pain – Discomfort PEO | 3.9 ± 2.5 | 3.9 ± 2.4 | 4.2 ± 2.9 |

*Values are means ± SD; groups did not differ significantly for any of the variables (p>0.05).*
